# Clinical Practice Guidelines for Rare Diseases: The Orphanet Database

**DOI:** 10.1371/journal.pone.0170365

**Published:** 2017-01-18

**Authors:** Sonia Pavan, Kathrin Rommel, María Elena Mateo Marquina, Sophie Höhn, Valérie Lanneau, Ana Rath

**Affiliations:** 1 Inserm, US14 - Orphanet, Paris, France; 2 Orphanet Germany, Centre for Rare Diseases, Hannover Medical School, Hannover, Germany; 3 Orphanet Spain, CIBERER, Valencia, Spain; Mayo Clinic, UNITED STATES

## Abstract

Clinical practice guidelines (CPGs) for rare diseases (RDs) are scarce, may be difficult to identify through Internet searches and may vary in quality depending on the source and methodology used. In order to contribute to the improvement of the diagnosis, treatment and care of patients, Orphanet (www.orpha.net) has set up a procedure for the selection, quality evaluation and dissemination of CPGs, with the aim to provide easy access to relevant, accurate and specific recommendations for the management of RDs. This article provides an analysis of selected CPGs by medical domain coverage, prevalence of diseases, languages and type of producer, and addresses the variability in CPG quality and availability. CPGs are identified via bibliographic databases, websites of research networks, expert centres or medical societies. They are assessed according to quality criteria derived from the Appraisal of Guidelines, REsearch and Evaluation (AGREE II) Instrument. Only open access CPGs and documents for which permission from the copyright holders has been obtained are disseminated on the Orphanet website. From January 2012 to July 2015, 277 CPGs were disseminated, representing coverage of 1,122 groups of diseases, diseases or subtypes in the Orphanet database. No language restriction is applied, and so far 10 languages are represented, with a predominance of CPGs in English, French and German (92% of all CPGs). A large proportion of diseases with identified CPGs belong to rare oncologic, neurologic, hematologic diseases or developmental anomalies. The Orphanet project on CPG collection, evaluation and dissemination is a continuous process, with regular addition of new guidelines, and updates. CPGs meeting the quality criteria are integrated to the Orphanet database of rare diseases, together with other types of textual information and the appropriate services for patients, researchers and healthcare professionals in 40 countries.

## Introduction

Clinical practice guidelines (CPGs) are "systematically developed statements to assist practitioners and patient decisions about appropriate health care for specific circumstances" [1]. Many CPGs have been developed in the last 25 years, as depicted in a recent review [2]. However most of them are aimed at common diseases and recommendations dedicated to rare diseases (RDs) remain scarce. In the European Union, a disease is considered as rare when it affects not more than 5 per 10,000 persons [3]. The rarity of randomised controlled trials puts a brake on the development of high quality guidelines for RDs, as well as the high development costs for funders who consider more prevalent diseases to be priority investment targets. In spite of these hurdles, over the last few years the development and dissemination of CPGs for RDs has garnered increasing attention [4–6]. The contribution of CPGs to shortening the time to diagnosis and improvement of the quality of care is now widely acknowledged, and several European countries have included CPG development as a priority in their respective national plans on RDs [7].

Retrieving RD guidelines from Internet searches is sometimes challenging for health professionals and patients who may not have the time or skills to search for the most relevant information [8]. Large national and international databases gathering CPGs are available [9–12] but they generally contain very few guidelines specific to RDs, which are difficult to find amongst the mass of recommendations available for more frequent diseases. In addition, in the field of RDs, a proportion of the guidelines produced by research networks, reference centres or other expert organisations is not published in international peer-reviewed journals, and thus is not retrieved from biomedical literature databases. Alternatively, Google searches may help identify RD guidelines, but the specificity of the suggested results is very low [13].

Once CPGs have been identified, another issue (not specific to RDs) might be their variable quality. To assess the quality of CPGs, several manuals have been published [14, 15]. One of the most employed and internationally validated grading systems is AGREE (Appraisal of Guidelines, Research and Evaluation) [16] and its revised version AGREE II Instrument [17]. Despite the availability of appraisal handbooks, methodological quality remains variable [2, 14, 18, 19] and needs to be verified. This might be even more important for RDs, as CPGs are sometimes developed by specialist groups who may not have the same resources as governmental bodies [2]. Methodologies for CPG development have been proposed that take into account some of the specificities of RDs, *e*.*g*. the lack of sound evidence, data about patients’ opinion, ethical considerations and surveys of clinical practices [5, 6, 20–22].

Orphanet is an international data resource dedicated to RDs that was created in 1997 to address the scarcity and fragmentation of information on RDs. It is co-funded through the European Union’s Health Programme and comprises a network of 40 countries. Orphanet endeavours to provide the community at large with a comprehensive set of information and data on RDs and orphan drugs in order to contribute to the improvement of the diagnosis, care and treatment of patients with RDs.

Orphanet is currently the most comprehensive repertoire of information and data on RDs, notably in terms of referenced documents, and the only resource that establishes a link between a classification of RDs, textual information and the appropriate services for patients, researchers and healthcare professionals.

This article describes the Orphanet workflow for the identification, evaluation and dissemination of CPGs on RDs, and provides an analysis of the resulting CPGs disseminated through Orphanet in terms of medical domain coverage, prevalence of diseases, languages and type of producer. Moreover, this study provides an insight into the variability in CPGs quality and availability, and shows the way these problems have been addressed in order to provide access to relevant, specific and good-quality recommendations for the management of RDs.

## Methods

### CPG identification and selection

**Fig 1:**
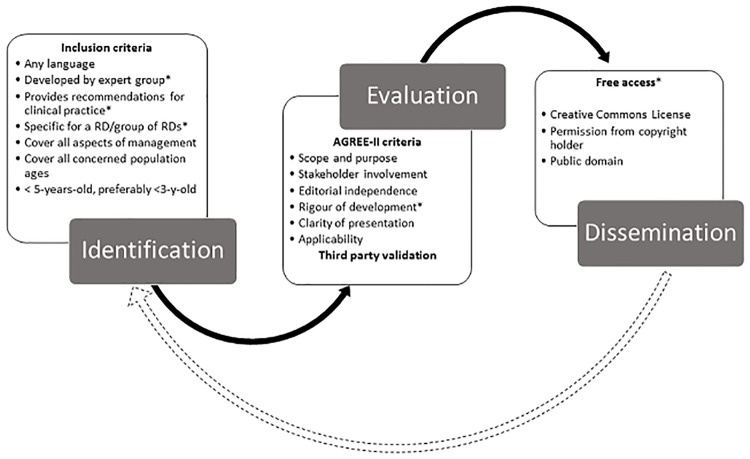
Methodological flow chart for CPG dissemination via Orphanet. * indicates the mandatory criteria versus the desirable ones for which exceptions can be made depending on all the other criteria.

Documents are considered as guidelines when they provide recommendations for clinical practice, in the form of consensus statements/recommendations, best practice statements or guidance recommendations. Only documents developed by expert groups are selected. Reviews providing some recommendations as concluding remarks or authors’ point of views are excluded, as well as recommendation documents released by single authors. A global bibliographic survey on RD literature is performed as a continuous process using a list of selected medical journals, which allows a small number of CPGs to be retrieved. It is completed by more specific bibliographic searches for guidelines and RDs on PubMed, Google and Google Scholar, each search engine often generating different and complementary search results. For certain diseases, websites of national and international databases, research networks, foundations, learned societies, governmental institutions and expert centres are browsed if applicable. Pearl-growing (using one resource to further identify other resources) is also frequently used. Although the most frequent sources of information are in English and in other major European languages, no language restriction is applied. Documents older than 5 years are not considered unless recent publications clearly state that they are still up-to-date. Preferably, recommendations less than three years-old are selected. Of note, some CPGs older than 5 years may still be up-to-date in spite of the absence of recent publication that states so. The 5-year selection limit may therefore result in the rejection of up-to-date recommendations. This cut-off was chosen as it is often applied by national guideline producers [23–25]. The topic should be directly related to a disease listed in the Orphanet nomenclature of RDs, and for dissemination purposes, when several CPGs are available, it was decided to include only the most comprehensive one. The topics covered are also taken into account: recommendations should cover almost, if not all aspects of disease management (*e*.*g*. guidance addressing only surgical intervention for skeletal anomalies in Marfan syndrome or on the use of a particular drug are excluded, guidelines proposing recommendations for only neurological issues for a multisystemic disease are not selected, etc.), unless a more general CPG is not available for this disease. Attention is paid to the universality of application of the guidelines with regard to the document language. For instance, guidelines in English that propose recommendations for inhabitants in China only will not be retained, but guidelines in Chinese that target Chinese patients will be considered. For the same reason, local CPGs are excluded. CPGs discussing recommendations for all population ages are preferred over CPGs targeting only children for a disease affecting all ages. Moreover, as much as possible, CPGs specific for rare forms of diseases are selected. However, due to the scarcity of available information, more general guidelines that also include recommendations for specific forms are sometimes retained (*e*.*g*., guidelines dealing with some cancers including both rare and non-rare forms). In summary, the quality evaluation procedure includes some mandatory criteria and some desirable ones (Fig 1), the latter being considered together with all the criteria in order to validate the decision to retain a document.

### Evaluation and dissemination

In addition to the specific criteria mentioned above, selected documents are evaluated by an information scientist according to quality criteria derived from the AGREE II Instrument, comprising 23 items organised into six domains: scope and purpose, stakeholder involvement, rigour of development, clarity of presentation, applicability and editorial independence [17]. To better fit the evaluation process to the actual guideline quality, the original rating system of AGREE II that uses a 7-point scale for each item (from strongly disagree to strongly agree) was simplified by yes/no answers. Appraisal of the six domains is carried out, but methodological aspects (domain 3 of AGREE II) are given particular weight: a poor methodological description leads to guideline rejection even if all other domains are outstanding. As in AGREE II, the assessors are asked whether they would recommend the use (here adapted into whether they would recommend dissemination) of the guideline. Then, overall assessment is carried out by taking into account the combined AGREE II and Orphanet inclusion criteria (Fig 1). Evaluated guidelines are validated by a third party (medical doctor) before the decision to display via Orphanet is taken. Authorisation from the copyright holders to disseminate CPGs is a key condition to the dissemination of guidelines via Orphanet. Articles published in “Open Access” (or with equivalent labelling: Open, Unlocked, …) and associated with a Creative Commons (CC) licence are directly made available without the need for specific permission. For documents that do not expressly state that free dissemination for non-commercial purposes is authorised, permission to establish links on www.orpha.net are first obtained from the copyright holder.

### Analysis of CPGs in the Orphanet database

The distribution of CPGs included in the Orphanet database until July 2015 was analysed by medical specialty. To do so, the linearisation of the RDs multi-hierarchical classification produced by Orphanet [26] was used. It provides a mono-hierarchical system in which each disease, usually included in multiple classifications, is allocated to a predominant body system. Specialties with no CPG were not displayed in the results. For CPGs associated with several Orphanet entries (CPGs targeting a disease with several subtypes, or a group of diseases), only the main target of the guideline was considered. Analyses of the publication languages were also carried out, either globally or by medical speciality. A Venn diagram representation [27] was used to visualise the diseases covered by CPGs in several languages.

The type of publication medium was analysed by distinguishing CPGs published in peer-reviewed journals and CPGs published by various organisations, independently of the type of authors.

Moreover, to analyse the CPG distribution as a function of disease prevalence, the Orphanet epidemiology database was used to retrieve the corresponding prevalence. As above, for CPGs associated with several entries (CPGs targeting a disease with several subtypes, or a group of diseases), only the main target of the guideline was considered for the choice of prevalence. The point prevalence was selected, and the geographic area corresponding to worldwide prevalence was used. When worldwide prevalence data were not available, Europe prevalence data were considered, then specific countries prevalence data (which was the case for only 2 diseases, for which USA prevalence was available). Diseases with no available prevalence data were not included in the analysis, which accounted for 44% of the diseases (22% with unknown prevalence and 22% with data not yet available in the Orphanet database). Diseases with CPGs in several languages were counted only once. Results are presented by class of prevalence, from the most frequent (1–10/10,000) to the rarest diseases (>1/1,000,000). Finally, an analysis of the number of CPGs downloaded in 2015 (1 January to 31 December) was carried out using Google analytics data. This analysis could be performed only on PDFs hosted on the Orphanet server, which accounted for 85 documents (31% of the disseminated CPGs). Audience analysis of the remaining 69% of guidelines was not possible due to their dissemination through direct links to the publishers’ websites.

## Results

### Quality evaluation of CPGs

Very few RD guidelines were found to meet a majority of the AGREE II criteria. Among the least followed criteria, we often noticed insufficient information about the management of conflicts of interest, insufficient information about the methodology to establish recommendations, a lack of consideration of patients’ preferences, and a lack of information about implementation, dissemination and updating procedures. Moreover, we observed variable quality depending on the dissemination medium. In general, guidelines disseminated in peer-reviewed journals were of better methodological quality than guidelines disseminated on websites of research networks or expert groups. For the latter, methodological procedures were often either incomplete, difficult to locate (presented in separate documents with no link provided in the guideline document) or missing.

In practice, depending on the scarcity of disease information, difficulty to retrieve the guidelines from Internet searches, recommendation quality, usefulness of specific information for health professionals, and the presence or absence of other documents on Orphanet covering the same topics, some exceptions were made to the coverage and target population inclusion criteria, to enable selection of relevant medical recommendations even when all disease aspects or target populations were not covered. In conclusion, limitations encountered at the CPG identification step led us to make some adjustments to the criteria used to retain or not a guideline.

### Guideline representation on Orphanet

**Fig 2:**
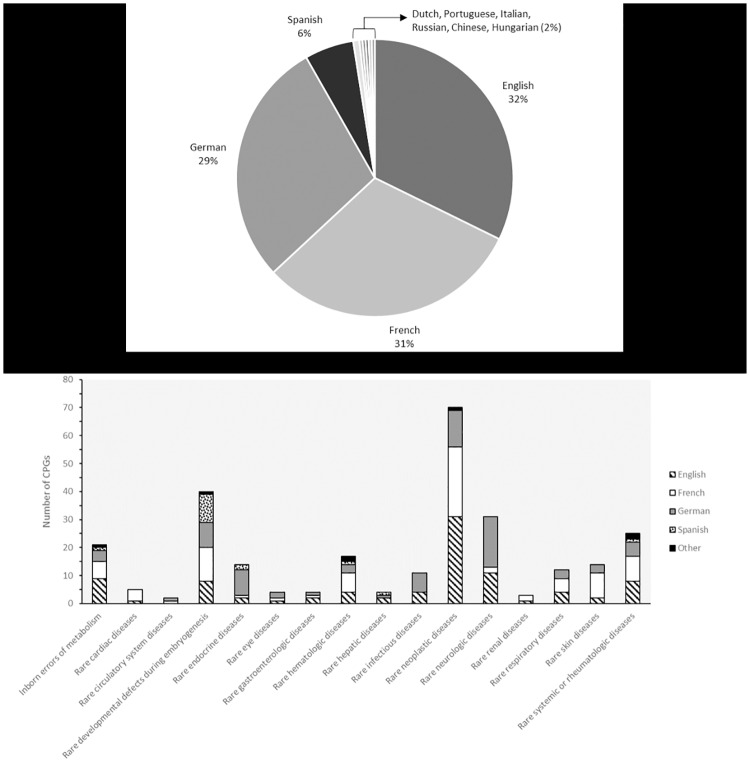
CPG distribution on Orphanet. (A) Proportion of CPGs available by language, (B) CPG distribution by medical specialty and language.

From January 2012 (beginning of this project) to July 2015, 277 CPGs were disseminated on www.orpha.net (S1 Table). They represent about half of the documents that fulfilled the content and quality criteria, the other half being rejected due to the impossibility to obtain copyright holders’ permissions to make links. Guidelines targeting several closely related diseases, or diseases that include etiological, histopathological or clinical subtypes were linked to each of the related diseases/subtypes in the database, which represents coverage of 1,122 entities. Ten languages were present, with a predominance of guidelines in English (32%), French (31%) and German (29%) (Fig 2A). About 6% of all texts were CPGs in Spanish, and the other 2% gathered CPGs in Hungarian, Italian, Dutch, Portuguese, Chinese and Russian.

Distribution of CPGs among medical specialties showed some “privileged” groups. Rare neoplastic diseases, neurologic, hematologic, rheumatologic and developmental diseases were the most represented, often with CPGs in both English, French and German (Fig 2B). It should be noted that, in the case of guidelines targeting multisystem diseases, the use of the linearised classification did not allow the involvement of a CPG covering multiple body systems to be shown. However, the use of this linearisation was chosen for the analysis in order to provide a combined picture of the exact number of CPGs, together with their distribution by medical specialty and by language of publication.

**Fig 3:**
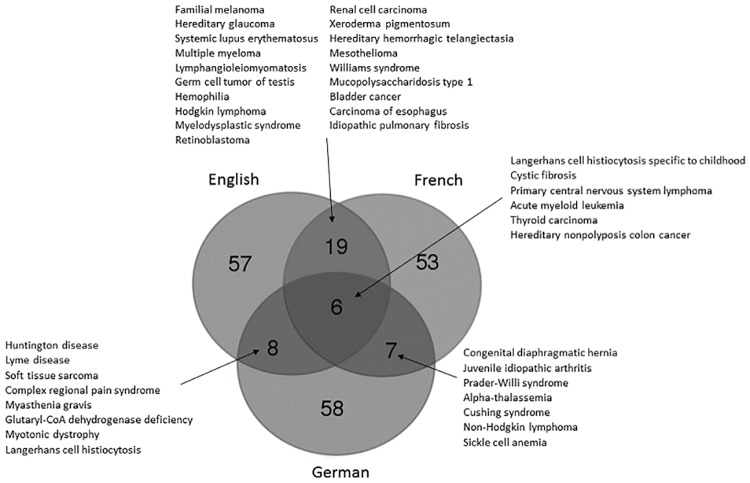
Repartition among diseases of CPGs in different languages. The Venn diagram representation shows the diseases covered by either one, two or three languages (English, French, German).

Among the French guidelines, 27% were from the French National Cancer Institute (INCa) [28], which, in addition to cancer guidelines from other sources, results in a strong representation of guidelines for neoplastic diseases. For CPGs in English, a more diverse range of author sources is observed because they were mostly published in medical journals, although guidelines on cancers were also clearly overrepresented (34% of all CPGs in English). Interestingly, very few diseases had guidelines simultaneously linked in several languages (Fig 3). Twenty-five diseases had CPGs in both English and French, 14 diseases had CPGs in both English and German, and 14 in both German and French. Only 6 diseases had CPGs in English, French and German.

**Fig 4:**
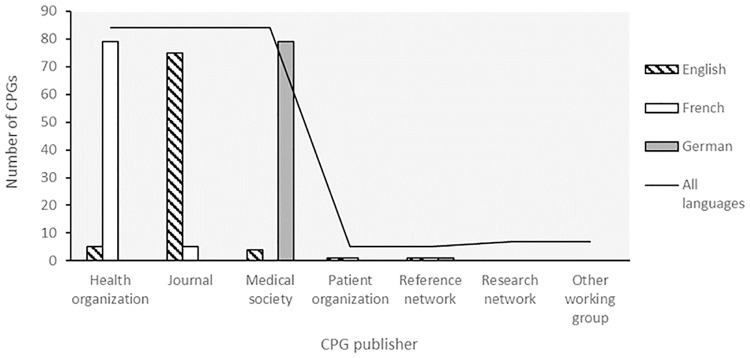
CPG distribution by type of publication medium and language.

Types of dissemination medium of CPGs differed depending on the language (Fig 4). CPGs in English were mostly published in international journals, and were most frequently authored by international working groups. The majority of CPGs in French were from public health organisations: the French National Authority for Health (HAS) and the INCa. The main sources of German guidelines were published by the Association of the Scientific Medical Societies in Germany (AWMF). Besides these three major publishers, a few of the collected guidelines were directly published by patient organisations, research networks, reference networks and other working groups on their websites. It should be mentioned that in all cases the CPGs were authored by expert working groups (which is one of the selection criteria), independently of the publication type.

**Fig 5:**
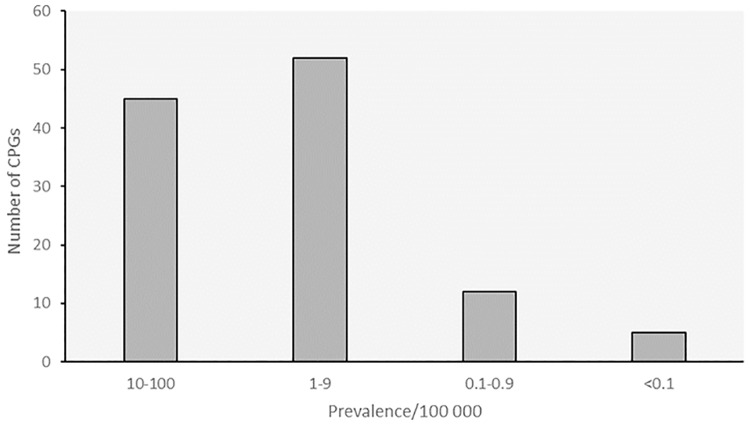
CPG distribution by disease prevalence.

About 20% of guidelines were related to diseases with higher prevalence (10–100 /100,000), while guidelines for extremely rare diseases were scarce (2% targeted diseases with prevalence <0.1/100,000) (Fig 5). In fact, some of these CPGs also include recommendations for rarer forms of the target diseases, with which they were also associated in the Orphanet database, but which were not taken into account in the analysis because only the main target of the guideline was considered for the choice of associated prevalence. Inclusion of these rarer forms in the analysis resulted in a slight shift of the number of CPGs towards diseases with lower prevalence, with about twice the amount of guidelines concerning diseases in the class of prevalence 1–9/100,000 (data not shown). Nevertheless, this shift did not impact the lowest class of prevalence (<0.1/100,000), which remained underrepresented.

### Access to the guidelines on Orphanet

Guidelines on a specific disease can be found by accessing the disease page via the disease search tool on www.orpha.net. A link “Clinical practice guidelines” appears in the “detailed information” section at the bottom of the disease page when a CPG is available. Moreover, the presence of guidelines can be visualised and accessed via the “Encyclopaedia for professionals” tab, where an alphabetical list of diseases and documents linked to them is displayed.

In 2015, more than 1,334,000 downloads of PDF files (concerning 31% of the guidelines in the database) were recorded, with a monthly average of >111,000 downloads. Since 2012, an increasing number of documents have been disseminated through links to the publishers’ websites and could not be included in the audience analysis. The PDF documents hosted on the Orphanet server thus represented the oldest guidelines and it is likely that these figures have underestimated the real number of consultations of all the guidelines available via Orphanet.

## Discussion

The difficulty to retrieve RD guidelines from the abundance of recommendations available for non-rare diseases is well known [13, 20]. As an additional challenge, the Orphanet database comprises more than 7,800 diseases/groups of diseases (excluding disorder subtypes) that represent potential targets for guideline retrieval, and to which only a global literature survey for guidelines and RDs can be applied, yielding very few appropriate results. Some medical societies and health organisations with websites gathering guidelines for a large variety of diseases, including RDs, were useful sources. In all cases, the retrieval of a potentially interesting document through this global survey was followed by specific disease guideline search to determine whether it was the most relevant CPG. Due to the diversity of publication media, it is clear that case-by-case approaches should be applied rather than a single retrieval strategy. Moreover, different search engines may be complementary to each other (*e*.*g*. Pubmed and Google Scholar) and may help in retrieving different documents. This is likely due to differences in the search algorithms rather than in differences in content coverage [29, 30].

With the aim to provide a comprehensive set of information and data on RDs to contribute to the improvement of the diagnosis, care and treatment of patients, the quality control of CPGs disseminated via the Orphanet database is an important aspect to consider. Therefore, we have set up an optimised evaluation procedure to provide sufficiently trustworthy information, without applying criteria that are too strict and that would lead to the rejection of documents containing nonetheless useful information. This is of particular importance with RDs, for which clinical trial data are sometimes insufficient compared to the data available for common diseases. Based on the AGREE II criteria, some insufficiencies were frequently noticed, especially regarding rigour of development and editorial independence. This observation has been described by others for some RDs [31] but is not specific to this field, as also reported for more common diseases [2, 32, 33]. Variable quality was also noticed depending on the publication medium. This discrepancy may reflect a lack of methodological expertise of guideline developers or limited resources. While it does not preclude the high medical quality of the recommendations, insufficient or lacking methodological data may impair the credibility of these guidelines. To overcome issues with variable quality, some national health institutions and medical societies have established standardisation procedures. This is the case of the HAS that has established within the first French National Plan for RDs a procedure based on the AGREE II criteria to evaluate the methodological rigour and transparency during the guideline elaboration process [23]. This methodology is implemented in the French National Diagnostic and Treatment Protocols (PNDS guidelines) for RDs. AWMF has adopted a similar procedure based on DELBI, The German Instrument for Methodological Guideline Appraisal (AGREE II-derived) in order “to provide a tool for the scientific medical societies to create and publish up-to-date and high-quality guidelines” [24]. The discrepancy in CPG quality explains the differences in the origin of publishers represented on Orphanet according to the language, and is the direct result of the applied quality evaluation process.

No language restriction was applied but a human resources factor accounted for a bias in guideline collection in various languages. Guidelines published in less spoken languages were often fortuitously collected, rather than actively searched for. Other factors also contributed to this imbalanced language representation: impossibility of obtaining permission to make links, insufficient methodological quality of guidelines, and last but not least, the non-existence of CPGs for many countries. For some countries in Europe, the development of guidelines and their diffusion have been set-up as a major objective of National Plans or strategies for RDs, in order to improve the quality of care for RD patients [7]. The inclusion of such objectives in the French and German National Plans also explains the high number of CPGs in French and German, in addition to CPGs in English that are most often the result of international working group publications in medical journals. A very low simultaneous coverage of diseases with guidelines in several languages was observed. This result underlines the importance of not applying language restrictions, as it allows larger guideline distribution among diseases.

Some recent initiatives to collect and disseminate RD guidelines are under development. RARE-BestPractices is an EU-funded project aiming to create a platform to share management guidelines for RDs [34]. It also aims at training stakeholders to produce and evaluate CPGs. CPGs indexed in this database undergo a quality validation process. Appraisal results are provided, but no filter based on the level of quality is applied for dissemination, and direct access to the document is not always provided, depending on the copyright conditions. The ZIPSE project aims to create an information portal for rare diseases in Germany to link existing information sources [35]. Among the collected information, links to guidelines and other documents disseminated through the Orphanet website shall be made. The Orphanet project on the collection, evaluation and dissemination of CPGs responds to the objectives of the EU’s Third Health Programme (2014–2020), in particular those concerning rare diseases [36], and to the Council recommendation on an action in the field of rare diseases of 8^th^ June 2009 that proposes to use Orphanet as a tool for information and research in order to contribute to improving the quality of care of patients, by improving the practice of health professionals and make information accessible through dissemination [37].

In addition to data quality assessment, free and direct (in one or two clicks) access to the linked information is an essential aspect of Orphanet’s editorial policy. Authorisation from the copyright holders to disseminate CPGs is mandatory, even when these documents are freely accessible (*i*.*e*. viewable but not reusable). For research networks (mostly EU-funded) and national health institutes, it is often not an issue, as the aim of these organisations is to freely disseminate CPGs, and linking guidelines on Orphanet contributes to increasing their diffusion and thus, achieving their goal. Permission from medical societies are usually more difficult to obtain, and document access is sometimes restricted to members only. For articles published in medical journals, granting of permission by publishers to make links free of charge constitutes the major limitation to broader guideline dissemination. The articles in question represent at least half of the potentially interesting CPGs. Therefore, the major proportion of journal articles linked on Orphanet consists in documents published with a CC licence allowing diffusion without the need for specific permission. Of note, the number of articles with a CC licence has considerably increased during the last few years, which allows more CPGs to be disseminated. In addition to articles reusable without permission, some publishers may grant specific permission to link an article freely, but this concerns a very limited number of CPGs. All the other articles cannot be disseminated on Orphanet due to the impossibility of obtaining the permission to make a link, irrespective of their quality. A complementary source of permission to disseminate CPGs resides in Orphanet’s active participation in the *patient* Inform collaborative program [38]. Indeed, through the production of disease summaries based on information contained in CPG articles, Orphanet is allowed to make special links to the articles, provided by the participating publishers. This allows patients and their caregivers to freely access relevant journal articles. Remarkably, while CPGs are originally dedicated to health professionals, a growing interest from patients and families for access to professional information is observed, that has paralleled the easier access to information provided by the Internet [39]. Empowerment of patients is also largely encouraged by their professional caregivers, through patient education [40].

Differences in the coverage of medical specialties were observed. Keeping in mind that guideline representation on Orphanet cannot be fully representative of all existing guidelines on RDs due to the applied selection criteria and non-exhaustive guideline retrieval, one can however make some parallels with the orphan drug development situation. When looking at the distribution of the number of drugs with orphan designation and authorisation on the market, antineoplastic agents represent 40% of all drugs [41]. Fifty-six percent of the ongoing clinical trials registered in the Orphanet database were on rare cancers. Also observed for CPGs in general (not only for RDs), the availability of guidelines is related to conditions for which more research and investment are made [2]. In the oncology field, the availability of guidelines is likely correlated with the existence of funding programs. At the European Commission level, a European Partnership on action against cancer (EPPAC) was created in 2009 aimed at the promotion of National cancer plans in EU Member States [42]. The EU-funded RareCareNet was set to build an information network on rare cancers [43]. In France, the INCa recommendation guides on cancers are elaborated within the framework of the National Cancer Plan in order to define and disseminate the reference clinical practices to health professionals and to the public [44]. In addition, the higher representation of oncology CPGs is influenced by the fact that some of these documents do not specifically address recommendations for RDs, but encompass both rare and non-rare entities. As observed in the field of non-rare diseases in which the availability of guidelines is correlated with higher prevalence [2], the distribution of RD guidelines via Orphanet is associated with relatively prevalent diseases: most CPGs concern diseases with prevalence between 5/10,000 and 9/100,000. This is likely due to the fact that the choice of diseases to be covered often takes into account the availability of a treatment or therapeutic solution (*e*.*g*. surgery) and the availability of clinical trial data. In the case of the French PNDS recommendations, for instance, prioritisation criteria for the choice of diseases to be covered include controversy over diagnosis, controversy over treatment, availability of new treatments and regular use of drugs without market authorisation [45].

## Conclusions

Access to information on RDs is one of the priorities adopted in most national plans in Europe [46]. Initiated in 2011, the Orphanet guideline dissemination project responds to this objective. In addition, it corresponds to a real need from both professionals and patients, as observed from our annual surveys [47] and from direct users’ feedback who have expressed their interest in accessing more clinical guidelines in more languages. CPG collection, evaluation and dissemination is a continuous process, with regular addition of new guidelines. Documents included in the Orphanet database may also be replaced by updates released by a guideline development group (providing they fulfil the quality criteria), old documents may be removed when they are obviously not up-to-date or according to the expiry date set by the development group, and finally, older guidelines may be replaced by more recent ones when appropriate, *e*.*g*. when a new document is of higher quality or when it is of similar quality but contains additional/more recent information useful for clinical practice. Compared to general guideline databases such as G-I-N [9], besides free access and quality assessment, CPGs disseminated via Orphanet are part of an information network gathered around the disease entity: each CPG can be accessed from the specific disease page where other types of information can be found, among which the ORPHA code, epidemiological and genetic data, disease summary, classification, health care resources, data on research activities and available drugs, links to other websites, guides for patients and families, etc. Therefore, the guideline database is included into a more global project that establishes a link between diseases, the existing textual information concerning RD and the appropriate services for patients, researchers and healthcare professionals. This integrative dimension is a unique feature of the Orphanet database that can serve various stakeholders and purposes. It can be used to identify medical areas for which CPGs are totally lacking but for which clinical research is active and/or drug development is ongoing.

## Supporting Information

S1 Table List of diseases associated with a clinical practice guideline disseminated on www.orpha.net. For each disease, the corresponding medical specialty considered for data analyses is indicated. “CPG language” refers to the language of publication of each CPG. “Source (publisher)” indicates in what medium the CPG has been published.
(PDF)
