# Supplementary material for: Clinical Practice Guidelines for Rare Diseases: The Orphanet Database
Source: PLoS One. 2017 Jan 18;12(1):e0170365. doi: 10.1371/journal.pone.0170365 (PMC5242437; doi:10.1371/journal.pone.0170365)
Supplement: S1 Table — For each disease, the corresponding medical specialty considered for data analyses is indicated. “CPG language” refers to the language of publication of each CPG. “Source (publisher)” indicates in what medium the CPG has been published. (PDF) [file pone.0170365.s001.pdf]

Disease	Medical specialty	CPG Language	Source (publisher)
Primary cutaneous anaplastic large cell lymphoma	Rare neoplastic disease	English	Journal article
Lymphangioleiomyomatosis	Rare respiratory disease	English	Journal article
Primary ciliary dyskinesia	Rare respiratory disease	English	Journal article
Idiopathic pulmonary fibrosis	Rare respiratory disease	English	Journal article
Homozygous familial hypercholesterolemia	Rare endocrine disease	English	Journal article
Rare cardiac rhythm disease	Rare cardiac disease	English	Journal article
Carcinoma of esophagus	Rare neoplastic disease	English	Journal article
Chronic autoimmune hepatitis	Rare hepatic disease	English	Journal article
Pancreatic endocrine tumor	Rare neoplastic disease	English	Journal article
Cholangiocarcinoma	Rare neoplastic disease	English	Journal article
Hereditary nonpolyposis colon cancer	Rare neoplastic disease	English	Journal article
Lyme disease	Rare infectious disease	English	Journal article
Congenital muscular dystrophy	Rare neurologic disease	English	Journal article
Nemaline myopathy	Rare neurologic disease	English	Journal article
Hereditary hemorrhagic telangiectasia	Rare developmental defect during embryogenesis	English	Journal article
Familial adenomatous polyposis	Rare gastroenterologic disease	English	Journal article
Desmoplastic small round cell tumor	Rare neoplastic disease	English	Journal article
Endocrine tumor	Rare neoplastic disease	English	Journal article
Malignant sex cord stromal tumor of ovary	Rare neoplastic disease	English	Journal article
Pancreatoblastoma	Rare neoplastic disease	English	Journal article
Thyroid carcinoma	Rare neoplastic disease	English	Journal article
Gastrointestinal stromal tumor	Rare neoplastic disease	English	Journal article
Primary hyperoxaluria type 1	Rare renal disease	English	Journal article
Juvenile dermatomyositis	Rare systemic or rheumatologic disease	English	Journal article
Gorlin syndrome	Rare developmental defect during embryogenesis	English	Journal article
Rare vascular liver disease	Rare hepatic disease	English	Journal article
Essential thrombocythemia	Rare hematologic disease	English	Journal article
Multiple myeloma	Rare neoplastic disease	English	Journal article
Hairy cell leukemia	Rare neoplastic disease	English	Journal article
Thrombotic thrombocytopenic purpura	Rare hematologic disease	English	Journal article
Chronic inflammatory demyelinating polyneuropathy	Rare neurologic disease	English	Journal article
Myasthenia gravis	Rare neurologic disease	English	Journal article
Focal, segmental or multifocal dystonia	Rare neurologic disease	English	Journal article
Autosomal recessive hypohidrotic ectodermal dysplasia	Rare developmental defect during embryogenesis	English	Journal article
Erdheim-Chester disease	Rare systemic or rheumatologic disease	English	Journal article
Central nervous system primitive neuroectodermal tumor	Rare neoplastic disease	English	Medical society
Primary central nervous system lymphoma	Rare neoplastic disease	English	Medical society
Astrocytoma	Rare neoplastic disease	English	Medical society

Hereditary glaucoma	Rare eye disease	English	Journal article
Q fever	Rare infectious disease	English	Health organization
Cystic fibrosis	Rare respiratory disease	English	Journal article
Langerhans cell histiocytosis specific to childhood	Rare systemic or rheumatologic disease	English	Reference network
Zygomycosis	Rare infectious disease	English	Journal article
Mesothelioma	Rare neoplastic disease	English	Journal article
Dermatofibrosarcoma protuberans	Rare neoplastic disease	English	Journal article
Familial melanoma	Rare neoplastic disease	English	Journal article
Germ cell tumor of testis	Rare neoplastic disease	English	Journal article
Acute myeloid leukemia	Rare neoplastic disease	English	Journal article
Small cell lung cancer	Rare neoplastic disease	English	Journal article
Bladder cancer	Rare neoplastic disease	English	Journal article
Rare uterine cancer	Rare neoplastic disease	English	Journal article
Soft tissue sarcoma	Rare neoplastic disease	English	Journal article
Rare head and neck tumor	Rare neoplastic disease	English	Journal article
Hodgkin lymphoma	Rare neoplastic disease	English	Journal article
Renal cell carcinoma	Rare neoplastic disease	English	Journal article
Eosinophilic esophagitis	Rare gastroenterologic disease	English	Journal article
Myotonic dystrophy	Rare neurologic disease	English	Journal article
Huntington disease	Rare neurologic disease	English	Journal article
Glycogen storage disease due to glycogen debranching enzyme deficiency	Inborn errors of metabolism	English	Journal article
Glycogen storage disease due to acid maltase deficiency	Inborn errors of metabolism	English	Journal article
West syndrome	Rare neurologic disease	English	Journal article
Waldenström macroglobulinemia	Rare neoplastic disease	English	Journal article
Chylomicron retention disease	Rare endocrine disease	English	Journal article
Mucopolysaccharidosis type 2	Inborn errors of metabolism	English	Journal article
Disorder of urea cycle metabolism and ammonia detoxification	Inborn errors of metabolism	English	Journal article
Langerhans cell histiocytosis	Rare systemic or rheumatologic disease	English	Journal article
Methylmalonic acidemia with homocystinuria	Inborn errors of metabolism	English	Journal article
Friedreich ataxia	Rare neurologic disease	English	Journal article
Inherited epidermolysis bullosa	Rare skin disease	English	Journal article
Mucopolysaccharidosis type 1	Inborn errors of metabolism	English	Journal article
Tyrosinemia type 1	Inborn errors of metabolism	English	Journal article
Angelman syndrome	Rare developmental defect during embryogenesis	English	Other working group
Ankylosing spondylitis	Rare systemic or rheumatologic disease	English	Journal article
Gaucher disease type 1	Inborn errors of metabolism	English	Health organization
Glutaryl-CoA dehydrogenase deficiency	Inborn errors of metabolism	English	Journal article
Acquired hemophilia	Rare hematologic disease	English	Journal article
Hereditary angioedema	Rare systemic or rheumatologic disease	English	Journal article

Kabuki syndrome	Rare developmental defect during embryogenesis	English	Other working group
Systemic lupus erythematosus	Rare systemic or rheumatologic disease	English	Journal article
Noonan syndrome	Rare developmental defect during embryogenesis	English	Other working group
Myelodysplastic syndrome	Rare neoplastic disease	English	Health organization
Psoriatic arthritis	Rare systemic or rheumatologic disease	English	Journal article
Retinoblastoma	Rare neoplastic disease	English	Journal article
Williams syndrome	Rare developmental defect during embryogenesis	English	Other working group
Xeroderma pigmentosum	Rare skin disease	English	Health organization
Complex regional pain syndrome	Rare neurologic disease	English	Medical society
22q11.2 deletion syndrome	Rare developmental defect during embryogenesis	English	Journal article
Hemophilia	Rare hematologic disease	English	Patient organization
Dengue fever	Rare infectious disease	English	Health organization
Moyamoya disease	Rare neurologic disease	English	Journal article
Hereditary glaucoma	Rare eye disease	French	Journal article
Gestational trophoblastic disease	Rare neoplastic disease	French	Health organization
Hodgkin lymphoma	Rare neoplastic disease	French	Health organization
Gestational trophoblastic neoplasm	Rare neoplastic disease	French	Health organization
Familial melanoma	Rare neoplastic disease	French	Health organization
Hepatocellular carcinoma	Rare neoplastic disease	French	Health organization
Primary central nervous system lymphoma	Rare neoplastic disease	French	Health organization
Multiple myeloma	Rare neoplastic disease	French	Health organization
Germ cell tumor of testis	Rare neoplastic disease	French	Health organization
B-cell chronic lymphocytic leukemia	Rare neoplastic disease	French	Health organization
Carcinoma of esophagus	Rare neoplastic disease	French	Health organization
Hereditary diffuse gastric cancer	Rare neoplastic disease	French	Health organization
Acute myeloid leukemia	Rare neoplastic disease	French	Health organization
Glanzmann thrombasthenia	Rare hematologic disease	French	Health organization
Classic Hodgkin lymphoma	Rare neoplastic disease	French	Health organization
Mesothelioma	Rare neoplastic disease	French	Health organization
Williams syndrome	Rare developmental defect during embryogenesis	French	Health organization
Familial atrial fibrillation	Rare cardiac disease	French	Health organization
Idiopathic nephrotic syndrome	Rare renal disease	French	Health organization
Hereditary nonpolyposis colon cancer	Rare neoplastic disease	French	Health organization
Idiopathic steroid-sensitive nephrotic syndrome	Rare renal disease	French	Health organization
Familial long QT syndrome	Rare cardiac disease	French	Health organization
Non-Hodgkin lymphoma	Rare neoplastic disease	French	Health organization
Nasopharyngeal carcinoma	Rare neoplastic disease	French	Health organization
Ovarian cancer	Rare neoplastic disease	French	Health organization
Hereditary breast cancer	Rare neoplastic disease	French	Health organization

Thyroid carcinoma	Rare neoplastic disease	French	Health organization
Bladder cancer	Rare neoplastic disease	French	Health organization
Renal cell carcinoma	Rare neoplastic disease	French	Health organization
Mayer-Rokitansky-Küster-Hauser syndrome	Rare developmental defect during embryogenesis	French	Health organization
Familial Mediterranean fever	Rare systemic or rheumatologic disease	French	Health organization
Congenital diaphragmatic hernia	Rare developmental defect during embryogenesis	French	Health organization
Familial medullary thyroid carcinoma	Rare neoplastic disease	French	Health organization
Gaucher disease	Inborn errors of metabolism	French	Health organization
Idiopathic and/or familial pulmonary arterial hypertension	Rare respiratory disease	French	Health organization
Mucopolysaccharidosis type 1	Inborn errors of metabolism	French	Health organization
Cystic fibrosis	Rare respiratory disease	French	Health organization
Autoimmune hemolytic anemia	Rare hematologic disease	French	Health organization
Medullar aplasia	Rare hematologic disease	French	Health organization
Juvenile idiopathic arthritis	Rare systemic or rheumatologic disease	French	Health organization
Esophageal atresia	Rare developmental defect during embryogenesis	French	Health organization
Bardet-Biedl syndrome	Rare developmental defect during embryogenesis	French	Health organization
CADASIL	Rare neurologic disease	French	Health organization
Cryopyrin-associated periodic syndrome	Rare systemic or rheumatologic disease	French	Health organization
Hypertrophic cardiomyopathy	Rare cardiac disease	French	Health organization
Tetralogy of Fallot	Rare developmental defect during embryogenesis	French	Health organization
Cushing syndrome	Rare endocrine disease	French	Health organization
Dermatitis herpetiformis	Rare skin disease	French	Health organization
Linear IgA dermatosis	Rare skin disease	French	Health organization
Fibromuscular dysplasia of arteries	Rare circulatory system disease	French	Health organization
Acquired epidermolysis bullosa	Rare skin disease	French	Health organization
Fabry disease	Inborn errors of metabolism	French	Health organization
Classic congenital adrenal hyperplasia due to 21-hydroxylase deficiency	Rare developmental defect during embryogenesis	French	Health organization
Pulmonary arterial hypertension	Rare respiratory disease	French	Health organization
Systemic lupus erythematosus	Rare systemic or rheumatologic disease	French	Health organization
Lymphangioleiomyomatosis	Rare respiratory disease	French	Health organization
Bullous pemphigoid	Rare skin disease	French	Health organization
Mucous membrane pemphigoid	Rare skin disease	French	Health organization
Pemphigoid gestationis	Rare skin disease	French	Health organization
Pemphigus vulgaris	Rare skin disease	French	Health organization
Phenylketonuria	Inborn errors of metabolism	French	Health organization
Prader-Willi syndrome	Rare developmental defect during embryogenesis	French	Health organization
Immune thrombocytopenic purpura	Rare hematologic disease	French	Health organization
Hereditary hemorrhagic telangiectasia	Rare developmental defect during embryogenesis	French	Health organization
Systemic sclerosis	Rare systemic or rheumatologic disease	French	Health organization

Stevens-Johnson syndrome	Rare skin disease	French	Health organization
Sickle cell anemia	Rare hematologic disease	French	Health organization
Myelodysplastic syndrome	Rare neoplastic disease	French	Health organization
Congenitally uncorrected transposition of the great arteries	Rare developmental defect during embryogenesis	French	Health organization
Truncus arteriosus	Rare developmental defect during embryogenesis	French	Health organization
Retinoblastoma	Rare neoplastic disease	French	Journal article
Alpha-thalassemia	Rare hematologic disease	French	Health organization
Turner syndrome	Rare developmental defect during embryogenesis	French	Health organization
Microscopic polyangiitis	Rare systemic or rheumatologic disease	French	Health organization
Xeroderma pigmentosum	Rare skin disease	French	Health organization
Idiopathic pulmonary fibrosis	Rare respiratory disease	French	Journal article
Medium chain acyl-CoA dehydrogenase deficiency	Inborn errors of metabolism	French	Journal article
Autosomal dominant non-syndromic intellectual disability	Rare neurologic disease	French	Journal article
Hemophilia	Rare hematologic disease	French	Patient organization
Langerhans cell histiocytosis specific to childhood	Rare systemic or rheumatologic disease	French	Reference network
Marfan syndrome	Rare systemic or rheumatologic disease	French	Health organization
Familial isolated hypertrophic cardiomyopathy	Rare cardiac disease	French	Health organization
Pediatric systemic lupus erythematosus	Rare systemic or rheumatologic disease	French	Health organization
Classic phenylketonuria	Inborn errors of metabolism	French	Health organization
MUTYH-related attenuated familial adenomatous polyposis	Rare gastroenterologic disease	French	Health organization
Asbestos intoxication	Rare respiratory disease	German	Medical society
Hirschsprung disease	Rare gastroenterologic disease	German	Medical society
Anorectal malformation	Rare developmental defect during embryogenesis	German	Medical society
Congenital diaphragmatic hernia	Rare developmental defect during embryogenesis	German	Medical society
Giant infantile hemangioma	Rare developmental defect during embryogenesis	German	Medical society
Hidradenitis suppurativa	Rare skin disease	German	Medical society
Ichthyosis	Rare skin disease	German	Medical society
Non-syndromic urogenital tract malformation of female	Rare developmental defect during embryogenesis	German	Medical society
Hereditary nonpolyposis colon cancer	Rare neoplastic disease	German	Medical society
Guillain-Barré syndrome	Rare neurologic disease	German	Medical society
Patent arterial duct	Rare developmental defect during embryogenesis	German	Medical society
Nephroblastoma	Rare neoplastic disease	German	Medical society
Osteosarcoma	Rare neoplastic disease	German	Medical society
Ewing sarcoma	Rare neoplastic disease	German	Medical society
Soft tissue sarcoma	Rare neoplastic disease	German	Medical society
Neuroblastoma	Rare eye disease	German	Medical society
Medulloblastoma	Rare eye disease	German	Medical society
Hepatoblastoma	Rare neoplastic disease	German	Medical society
Non-Hodgkin lymphoma	Rare neoplastic disease	German	Medical society

Langerhans cell histiocytosis	Rare systemic or rheumatologic disease	German	Medical society
Sickle cell anemia	Rare hematologic disease	German	Medical society
Alpha-thalassemia	Rare hematologic disease	German	Medical society
Hereditary spherocytosis	Rare hematologic disease	German	Medical society
Rare tumor of neuroepithelial tissue	Rare neoplastic disease	German	Medical society
Ependymal tumor	Rare neoplastic disease	German	Medical society
Acute myeloid leukemia	Rare neoplastic disease	German	Medical society
Mitochondrial disease	Inborn errors of metabolism	German	Medical society
Primary congenital hypothyroidism	Rare endocrine disease	German	Medical society
Glutaryl-CoA dehydrogenase deficiency	Inborn errors of metabolism	German	Medical society
Juvenile idiopathic arthritis	Rare systemic or rheumatologic disease	German	Medical society
Androgen insensitivity syndrome	Rare developmental defect during embryogenesis	German	Medical society
Malformation syndrome with short stature	Rare developmental defect during embryogenesis	German	Medical society
Precocious puberty	Rare endocrine disease	German	Medical society
Panhypopituitarism	Rare endocrine disease	German	Medical society
Congenital central diabetes insipidus	Rare endocrine disease	German	Medical society
Cushing syndrome	Rare endocrine disease	German	Medical society
Primary adrenal insufficiency	Rare endocrine disease	German	Medical society
Familial primary hyperparathyroidism	Rare endocrine disease	German	Medical society
Familial hyperthyroidism due to mutations in TSH receptor	Rare endocrine disease	German	Medical society
Thyroid carcinoma	Rare neoplastic disease	German	Medical society
Congenital isolated hyperinsulinism	Inborn errors of metabolism	German	Medical society
Adrenogenital syndrome	Rare endocrine disease	German	Medical society
Severe acute respiratory syndrome	Rare respiratory disease	German	Medical society
Amyotrophic lateral sclerosis	Rare neurologic disease	German	Medical society
Trigeminal neuralgia	Rare neurologic disease	German	Medical society
Huntington disease	Rare neurologic disease	German	Medical society
Tick-borne encephalitis	Rare infectious disease	German	Medical society
Cluster headache	Rare neurologic disease	German	Medical society
Rare dystonia	Rare neurologic disease	German	Medical society
Creutzfeldt-Jakob disease	Rare neurologic disease	German	Medical society
Mitochondrial myopathy	Rare neurologic disease	German	Medical society
Polymyositis	Rare systemic or rheumatologic disease	German	Medical society
Myotonic dystrophy	Rare neurologic disease	German	Medical society
Narcolepsy-cataplexy	Rare neurologic disease	German	Medical society
Primary central nervous system lymphoma	Rare neoplastic disease	German	Medical society
Rare peripheral neuropathy	Rare neurologic disease	German	Medical society
Lyme disease	Rare infectious disease	German	Medical society
Stiff person syndrome	Rare neurologic disease	German	Medical society

Giant cell arteritis	Rare systemic or rheumatologic disease	German	Medical society
Myasthenia gravis	Rare neurologic disease	German	Medical society
Meningococcal meningitis	Rare infectious disease	German	Medical society
Wilson disease	Rare hepatic disease	German	Medical society
Cerebral cavernous malformations	Rare developmental defect during embryogenesis	German	Medical society
Glial tumor	Rare neoplastic disease	German	Medical society
Encephalitis	Rare neurologic disease	German	Medical society
Tetanus	Rare infectious disease	German	Medical society
Botulism	Rare infectious disease	German	Medical society
Muscular dystrophy	Rare neurologic disease	German	Medical society
Complex regional pain syndrome	Rare neurologic disease	German	Medical society
X-linked adrenoleukodystrophy	Inborn errors of metabolism	German	Medical society
Limbic encephalitis	Rare neurologic disease	German	Medical society
Lipedema	Rare skin disease	German	Medical society
Malaria	Rare infectious disease	German	Medical society
Leishmaniasis	Rare infectious disease	German	Medical society
Lymphedema	Rare circulatory system disease	German	Medical society
Cystic fibrosis	Rare respiratory disease	German	Medical society
Fragile X-associated tremor/ataxia syndrome	Rare neurologic disease	German	Medical society
Prader-Willi syndrome	Rare developmental defect during embryogenesis	German	Medical society
Langerhans cell histiocytosis specific to childhood	Rare systemic or rheumatologic disease	German	Reference network
Langerhans cell histiocytosis	Rare systemic or rheumatologic disease	Spanish	Reference network
Cowden syndrome	Rare developmental defect during embryogenesis	Spanish	Other working group
Megalencephaly-capillary malformation-polymicrogyria syndrome	Rare developmental defect during embryogenesis	Spanish	Other working group
Bannayan-Riley-Ruvalcaba syndrome	Rare developmental defect during embryogenesis	Spanish	Other working group
Gaucher disease	Inborn errors of metabolism	Spanish	Research network
Alström syndrome	Rare developmental defect during embryogenesis	Spanish	Research network
Monosomy 5p	Rare developmental defect during embryogenesis	Spanish	Research network
Cushing syndrome	Rare endocrine disease	Spanish	Research network
22q11.2 deletion syndrome	Rare developmental defect during embryogenesis	Spanish	Research network
Leprechaunism	Rare endocrine disease	Spanish	Research network
Androgen insensitivity syndrome	Rare developmental defect during embryogenesis	Spanish	Other working group
Wolf-Hirschhorn syndrome	Rare developmental defect during embryogenesis	Spanish	Research network
WAGR syndrome	Rare developmental defect during embryogenesis	Spanish	Other working group
Hemophilia	Rare hematologic disease	Spanish	Patient organization
Progressive familial intrahepatic cholestasis type 2	Rare hepatic disease	Spanish	Other working group
Costello syndrome	Rare developmental defect during embryogenesis	Spanish	Other working group
Fabry disease	Inborn errors of metabolism	Hungarian	Journal article
Langerhans cell histiocytosis	Rare systemic or rheumatologic disease	Italian	Reference network

Noonan syndrome	Rare developmental defect during embryogenesis	Dutch	Other working group
Waldenström macroglobulinemia	Rare neoplastic disease	Dutch	Journal article
Systemic sclerosis	Rare systemic or rheumatologic disease	Portuguese	Journal article
Hemophilia	Rare hematologic disease	Russian	Patient organization
Hemophilia	Rare hematologic disease	Chinese	Patient organization
